# Phytohormone combined with nitrogen stress promoted carbon conversion in CO_2_ chemical absorption and microalgae conversion system

**DOI:** 10.3389/fbioe.2025.1661092

**Published:** 2025-09-03

**Authors:** Zhan Hu, Dantong Wang, Pengcheng Li, Yaoqi Hou, Guanyi Chen, Chunfeng Song

**Affiliations:** Tianjin Key Laboratory of Indoor Air Environmental Quality Control, School of Environmental Science and Engineering, Tianjin University, Tianjin, China

**Keywords:** CAMC system, *Dunaliella salina*, nitrogen stress, biomass, β-carotene

## Abstract

The CO_2_ chemical absorption and microalgae conversion (CAMC) system can achieve low-energy consumption CO_2_ capture and resource utilization, but the study on enhancing the synthesis of high-value products has been overlooked. In this study, the coupling strategy of nitrogen stress and exogenous phytohormones was applied to induce high-value products accumulation of *Dunaliella salina* in CAMC system, and the potential mechanism was also discussed. The results showed that phytohormones could promote *D. salina* growth by alleviating the oxidative damage under nitrogen limited treatment, especially in gibberellin (GA) group, and the biomass increased by 10.24%. In addition, phytohormones combined nitrogen limited changed carbon flow, directing more carbon to polysaccharose synthesis, supplementation with GA under nitrogen-limited conditions resulted in a 1.65-fold increase in polysaccharide content in D. salina compared to the control. Furthermore, supplementation with GA under nitrogen-limited conditions enhanced the accumulation of β-carotene, and the β-carotene yield and content were 18.52% and 14.46% higher than control. This study suggested that GA could further affect carbon metabolism and products synthesis of *D. salina* under nitrogen stress, and provided a possible insight in improving the production of economical metabolites in CAMC system.

## 1 Introduction

As one of the major greenhouse gases contributing to global warming, carbon dioxide (CO_2_) emission reduction has received widespread attention (Karimi et al., 2023). Carbon capture, utilization, and storage (CCUS) has been considered to a significant technology for achieving net-zero emissions (Lin et al., 2022). At present, biologically-mediated carbon capture technology using microalgae has been concerned due to its feasibility, sustainability and profitability (Daneshvar et al., 2022). Despite this potential, the high CO_2_ supply cost and low carbon utilization efficiency increase the microalgae cultivation cost and limit the microalgae CO_2_ fixation (Chi et al., 2011). Therefore, developing other forms of carbon source as feedstock for microalgae photosynthesis is crucial.

The existence forms of dissolved inorganic carbon in the medium are H_2_CO_3_, CO_2_, CO_3_^2−^ and HCO_3_^−^. Carbonic anhydrase (CA) in microalgae can realize the conversion from HCO_3_^−^ to CO_2_ (Li et al., 2018). Based on the ability of microalgae to utilize HCO_3_^−^, bicarbonate is proposed as an alternative carbon source (Li et al., 2018; Zhu et al., 2022). Therefore, the concept of CO_2_ chemical absorption and microalgae conversion system (CAMC) was proposed in previous study (Song et al., 2019a). In this system, the absorbent rich of bicarbonate after absorbing CO_2_ is used to culture microalgae (Song C. F. et al., 2019), realizing the transformation from CO_2_ to bicarbonate and then to microalgal biomass. Previous study also showed that supplementing bicarbonate could promote the accumulation of metabolites (Singh et al., 2025b). Therefore, the metabolites in microalgal biomass with high economic value, such as lipid, pigment and protein, bring economic benefits to the CAMC system. However, previous studies focused on enhancing the carbon sequestration performance of the CAMC system, but the research on enhancing the synthesis of high-value products by microalgae in the CAMC system has been overlooked.

*Dunaliella salina*, a single-celled green alga, has the characteristics of high pH tolerance and bicarbonate concentration tolerance (Zhu et al., 2022), which is suitable for the CAMC system. As its characteristic product, β-carotene is a valuable biochemical and has attracted great attention (Xi et al., 2020). Besides being a food pigment, β-carotene is served as vitamin A precursor and has antioxidative properties (Pourkarimi et al., 2020). It also has multiple beneficial effects on human health (Rammuni et al., 2019). Nowadays, *Dunaliella salina* has been considered as an appropriate option for extraction of valuable β-carotene due to its super ability of accumulating β-carotene (Pourkarimi et al., 2020; Xi et al., 2022). Therefore, it is necessary to culture *D. salina* in CAMC system and explore the strategy that inducing β-carotene accumulation to improve the economic feasibility of CAMC system.

In recent years, the coupling strategy of abiotic stresses and exogenous phytohormones were used for the production of high-valued products (Zhao et al., 2019b). Abiotic stress made microalgae in the state of oxidative stress and promoted the synthesis of metabolites, while phytohormone could promote microalgae growth and alleviate oxidative damage of microalgae cells. For example, Yang et al. (2023) showed that phytohormones could improve lipid synthesis, and the lipid production was 3.7 times than control. Li et al. (2024) showed that phytohormones could induced the carbon flux flow to the synthesis of carbohydrate and protein, and increased carbohydrate and protein productivity. As a common stress mean, nitrogen stress combined with phytohormones used to induce products accumulation has been reported (Singh et al., 2024a; Song et al., 2022). Singh et al. (2024b) showed that supplementation of IAA under nitrogen deprivation led to a significant rise in the lipid content (47% DCW), carbohydrate (18.37%). Yu et al. (2018) cultured microalgae under nitrogen depletion condition with the supplement of phytohormones and obtained higher lipid productivity. Nitrogen stress could activate antioxidant system and change the carbon flow of microalgae cells, and the application of phytohormones could further affect the carbon metabolism and expression of related genes (Zhao et al., 2018). However, the effect of nitrogen stress treatment combined exogenous phytohormones on the growth and product accumulation of *D. salina* under bicarbonate culture system (CAMC system) are not clear. Therefore, it is necessary to apply the culture strategy of nitrogen stress combined with phytohormones on *D. salina* in CAMC system, and investigate the effects of different phytohormones on microalgae growth and β-carotene accumulation under nitrogen stress.

In this study, *D. salina* were cultured in CAMC system under nitrogen stress conditions, indole butyric acid (IBA), gibberellin (GA) and abscisic acid (ABA) were supplemented during the culture period to explore the different response of microalgae to exogenous phytohormones under nitrogen stress. The microalgae growth, pigments productivity, characteristic product accumulation and metabolites content were evaluated. And the key enzyme (Rubisco) and metabolite (Acetyl-CoA) content involved in central carbon metabolism were tested to further illustrate the effect of the coupled treatment on the *D. salina*. In addition, the antioxidant enzyme activity (SOD and CAT) and nonenzymatic antioxidant (GSH) content were measured to explain the response of algae to stress conditions and the possible mechanisms of phytohormones to alleviate stress. This study illustrated the effect of stress treatment combined phytohormones on CAMC system, and provides a new possibility of further improving the CO_2_ resource utilization efficiency.

## 2 Materials and methods

### 2.1 Microalgae strain and culture

The microalga strain used in this study was *D. salina* FACHB-435, which was obtained from the Freshwater Algae Culture Collection at the Institute of Hydrobiology, (Wuhan, China). Before inoculation, *D. salina* was cultivated under autotrophic conditions with constant light supply in *Dunaliella* medium (Chen et al., 2024). The chemical composition of *Dunaliella* medium was as follows: nutrient solution (NaNO_3_: 0.42 g/L, NaH_2_PO_4_·2H_2_O: 0.0156 g/L, CaCl_2_·2H_2_O: 0.044 g/L, KCl: 0.074 g/L, MgSO_4_·7H_2_O: 1.23 g/L, NaHCO_3_: 0.84 g/L), trace mental solution (H_3_BO_3_: 2.86 mg/L, MnCl_2_·4H_2_O: 1.86 mg/L, ZnSO_4_·7H_2_O 1.22 mg/L, Na_2_MoO_4_·2H_2_O: 0.39 mg/L, CuSO_4_·5H_2_O: 0.08 mg/L, Co(NO_3_)_2_·6H_2_O: 0.05 mg/L), and saline solution (NaCl: 87.69 g/L). The average temperature was controlled at 26 °C ± 1 °C during the culture period.

### 2.2 Experimental design

In this study, bicarbonate in the rich absorption solution, which was generated after the absorption of CO_2_ by chemical absorbent, was supplied to microalgae as carbon source in CAMC system. The concentration of bicarbonate was 16.8 g/L and added to the *Dunaliella* medium. The microalgae were cultivated in 250 mL conical flasks and divided into five groups. In the first group (control group), the concentration of NaNO_3_ in *Dunaliella* medium was not changed, with 0.42 g/L. In the second group (Nitrogen limited group: NL group), the concentration of NaNO_3_ in *Dunaliella* medium was reduced to 0.1 g/L, in order to make the microalgae in the nitrogen limited state in the late stage of culture. The other three groups were supplemented with different phytohormones (IBA, GA, ABA) on the basis of the second group on the third day of the culture period. To evaluate the effects of these three phytohormones in the same concentration on cell growth and product accumulation, the concentration of the phytohormones was all set to 10 mg/L by the screening of the pre-experiment. The initial inoculation concentration of microalgae cells was controlled at approximately 0.2 (OD_680_). The cultivation was performed at 4,000 lx, and a light-dark ratio of 16/8. IBA, GA, ABA were purchased from Sigma-Aldrich (Shanghai, China), Aladdin (Shanghai, China), and Heowns (Tianjin, China), respectively. Each group had three parallel experiments, and the flasks were shaken every day to prevent aggregation.

### 2.3 Determination of microalgae biomass concentration and product content

#### 2.3.1 Determination of microalgae biomass concentration

The absorbance value of *D. salina* at 680 nm has a linear relationship with its dry weight, so the dry weight was calculated according to the standard curves of the optical density at 680 nm measured with UV-vis spectrophotometer and biomass dry weight. The standard curve was obtained as follows: A certain volume of algal liquid was filtered on a 0.45 µm filter paper that has been dried to constant weight in advance. Then the filter paper with microalgal cells was placed in a blast drying oven at 105 °C to a constant weight. The dry weight of alga was obtained by the weight difference.

#### 2.3.2 Determination of microalgae pigment content

The chlorophyll and carotenoids in fresh microalgae cells were extracted with methanol (90%, v/v) and placed in refrigerator at 4 °C for 20 h. The OD values at 470 nm, 652.4 nm, 665.2 nm were measured by ultraviolet spectrophotometry respectively, and the concentrations of chlorophyll a, chlorophyll b and carotenoid were calculated by Equations 1–3 (Xie et al., 2022):

$$\text{Chlorophyll }a\;\left(\text{mg}/L\right)=16.72\times A_{665.2}‐9.16\times A_{652.4}$$ (1)

$$\text{Chlorophyll }b\;\left(\text{mg}/L\right)=34.09\times A_{652.4}‐15.28\times A_{665.2}$$ (2)

$$\text{Carotenoids }\left(\text{mg}/L\right)=\left(1000\times A_{470}‐1.63\times C_{a}‐104.96\times C_{b}\right)/221$$ (3)

#### 2.3.3 Determination of β-carotene

The β-carotene concentration was determined by modified spectrophotometric method (Zhu et al., 2018). 1 mL cell suspension was centrifuged at 5,000 rpm for 10 min, and the supernatant was discarded. 3 mL dodecane was added to the obtained precipitate, and vortex oscillated to suspend the microalgae cells again. Then, 6 mL methanol was added to break the microalgae cells. After vortex oscillating again, the sample was centrifuging at 5,000 rpm for 10 min. The absorbance of the upper solution (dodecane layer) was measured at 453 nm and 665 nm. The β-carotene concentration in microalgae cells was calculated by the following Equation 4:

$$\beta ‐\text{carotene }\left(\text{mg}/L\right)=\left(A_{453}‐A_{665}/\;3.91\;\right)\times 3.657\times 3$$ (4)

#### 2.3.4 Determination of lipid, protein and polysaccharide content

In this work, the lipid content in microalgae cells was determined by Nile red staining (Song et al., 2021). The lipid concentration was calculated by Equation 5. Polysaccharide content was determined by anthrone sulfuric acid method, and the detail extracted measure was referred to previous study (Han et al., 2021). The polysaccharide concentration was calculated by the standard curve Equation 6. The total nitrogen content of freeze-dried microalgae was measured by elemental analysis, with reference to the previous literature (Song C. et al., 2019). The conversion factor of total nitrogen-protein was 6.25, and the calculation was shown as Equation 7.

$$\text{Lipid concentration }\left(\text{mg}/L\right)=\left({\text{OD}}_{580}+7.10\right)/1.98$$ (5)

$$\text{Polysaccharide concentration }\left(\text{mg}/L\right)=19.88\times {\text{OD}}_{620}+0.498$$ (6)

$$\text{Protein concentration }\left(\text{mg}/L\right)=\text{DW}\times N_{\text{alga}}\times 6.25$$ (7)

where, DW is the dry weight of microalgae (mg/L), N_alga_ is the proportion of nitrogen element in algae (%).

### 2.4 Determination of enzyme activity and oxidative stress biomarker

The enzyme activities of Rubisco, Superoxide Dismutase (SOD), and Catalase (CAT) were determined by Rubisco assay kit (RUBPS-2A-Y, Comin, Suzhou, China), SOD assay kit (SOD-2-Y, Comin, Suzhou, China), and CAT assay kit (CAT-2-Y, Comin, Suzhou, China), respectively. Acetyl-CoA content was measured with Acetyl-CoA assay kit (ACA-2A-Y, Comin, Suzhou, China). The GSH level was determined by GSH assay kit (GSH-2-W, Comin, Suzhou, China).

### 2.5 Statistical analysis

The data are presented as means ± standard deviation (SD, n = 3). The statistical analysis is carried out using SPSS 27 software. One-way analysis of variance (ANOVA) is conducted to identify the differences between the means, and p < 0.05 indicates significant difference. Each group had three parallel experiments.

## 3 Results and discussion

### 3.1 Effect of phytohormones on D. salina growth under nitrogen stress

**FIGURE 1:**
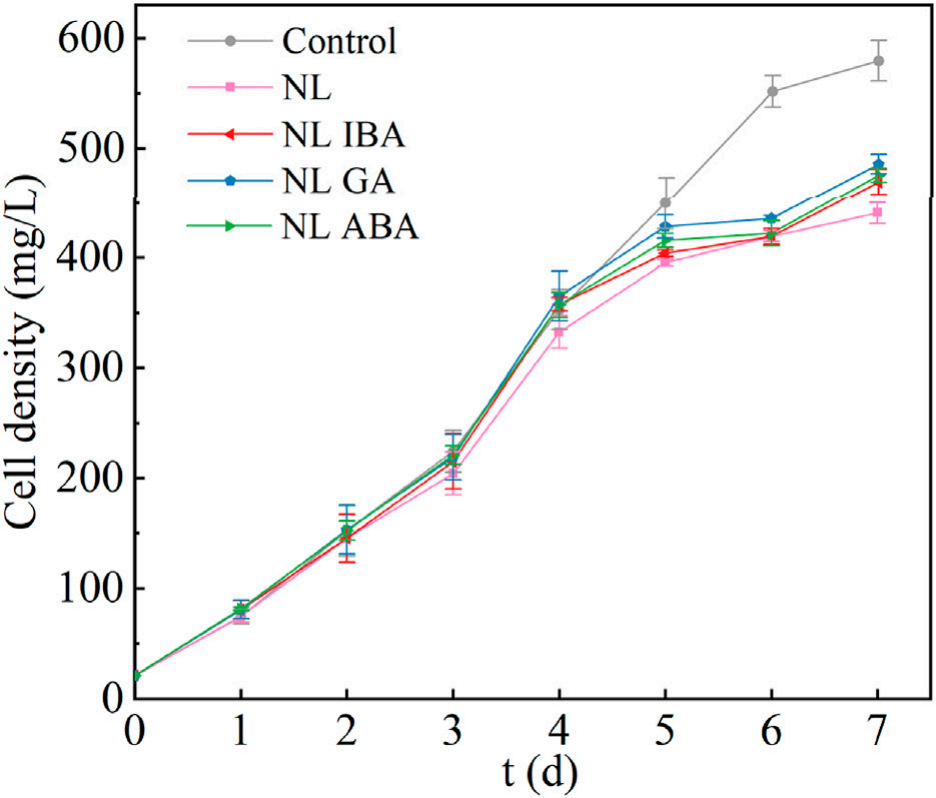
The biomass concentration of *Dunaliella salina* under different conditions.

The application of environmental stress strategies on the production of high-value-added products of microalgae also has the trade-off problem of biomass decline. The growth of *D. salina* under different conditions was monitored for 7 days, shown in Figure 1. It could be observed that nitrogen stress (NL group) directly inhibited the growth of *D. salina*, compared with the normal medium. It was notable that the application of phytohormones promoted microalgae growth under nitrogen limited treatment condition. At the end of the culture, the biomass concentration of IBA, GA and ABA group reached 469.66 ± 11.48 mg/L, 485.86 ± 8.91 mg/L and 475.73 ± 6.26 mg/L, which was 6.56%, 10.24% and 7.94% higher than that of the nitrogen limited group, respectively. This might be because phytohormones activated the antioxidant enzymes, which then removed the ROS within the cells and played a role in protecting the photosystem. On the other hand, phytohormones also played a role in regulating osmotic, ionic and redox balance, thereby helping to maintain the homeostasis of cells. Zhou et al. (2024) showed that phytohormones could promote the establishment of microalgal cell homeostasis, thereby resisting environmental fluctuations. Many studies have indicated that phytohormones could improve the resistance of microalgae to environmental stress, e.g., by promoting chlorophyll synthesis and increasing the activity of antioxidant enzymes, then promoting the growth of microalgae (Chang et al., 2022; Yang et al., 2023; Yu et al., 2023; Zhao et al., 2018). In this work, although the promoting effect of exogenous phytohormones did not completely offset the inhibitory effect of nitrogen stress. However, under nitrogen stress, the promotion of microalgae biomass by phytohormones may be conducive to increasing the yield of high-value-added products of microalgae and the resource utilization efficiency of CO_2_. Besides, the types of phytohormones also have different effects on *D. salina*, among which, GA had the best effect on promoting microalga growth at the same concentration (P < 0.05). To sum up, adding exogenous phytohormones was an effective strategy to increase biomass concentration of microalgae under stress conditions. Due to the different promoting level of phytohormones on microalgae, the type of phytohormones was also a factor that needed to be considered when applying the culture strategy of stress combined with phytohormones in CAMC system.

### 3.2 Change of pigment concentrations

**FIGURE 2:**
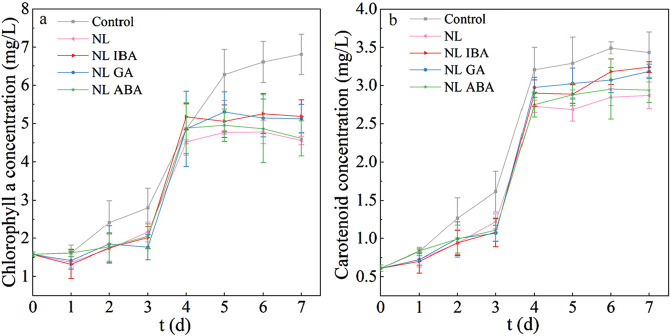
The pigment concentrations of *Dunaliella salina* under different culture conditions: **(a)** chlorophyll a; **(b)** carotenoids.

As the important pigments in microalgae cells, chlorophyll a and carotenoids played a curial role in photosynthetic reactions and served as a key physiological indicator (Wan et al., 2021). Figure 2a showed the variation of chlorophyll a content during the culture period. It could be observed that the chlorophyll a content of microalgae was decreased under nitrogen stress, compared with the normal nitrogen concentration. The explanation of this phenomenon was that the depletion of nitrogen in the medium might result in the partial decomposition of chlorophyll a which was composed of nitrogen. Liu Q. et al. (2024) also found that Nitrogen limitation would inhibit the synthesis of chlorophyll. Moreover, the addition of phytohormones increased the chlorophyll a content in nitrogen limited medium. Compared with the results observed in nitrogen limited group, the chlorophyll a concentration in the groups supplemented with IBA and GA reached 5.1 ± 0.43 mg/L and 5.12 ± 0.37 mg/L, which increased by 13.76% and 12.46%, respectively. This result was consistent with the better microalgae growth under phytohormones treatment in NL group (Figure 1). Previous study also reported that phytohormones promoted the total chlorophyll content under nitrogen-starved (Yu et al., 2018). This may be because that phytohormones could improve the resistance of microalgae to oxidative stress, and enhance the ability of cells to use intracellular N by increasing the activity of the cells, which contributed to protecting the organelle and maintained chlorophyll content under nitrogen limited condition.

Carotenoids played a role in protecting microalgae cells from oxidative damage (Jahns and Holzwarth, 2012). As shown in Figure 2b, the variation trend of carotenoid was similar to chlorophyll a. It was worth noting that the carotenoid content of *D. salina* exposed to IBA and GA in NL group reached 3.24 ± 0.07 mg/L and 3.18 ± 0.09 mg/L, respectively, and was higher than that of the NL blank group. The increased carotenoid content under the action of phytohormones helps to improve the antioxidant capacity and scavenge excess free radicals under nitrogen limited treatment. To sum up, this study suggested that IBA and GA could promote the accumulation of pigments (e.g., chlorophyll a and carotenoids) under nitrogen stress, and then promoted photosynthetic activity and protected cells from oxidative damage, which was benefit to promote *D. salina* growth in CAMC system.

### 3.3 Enzyme activity change

**FIGURE 3:**
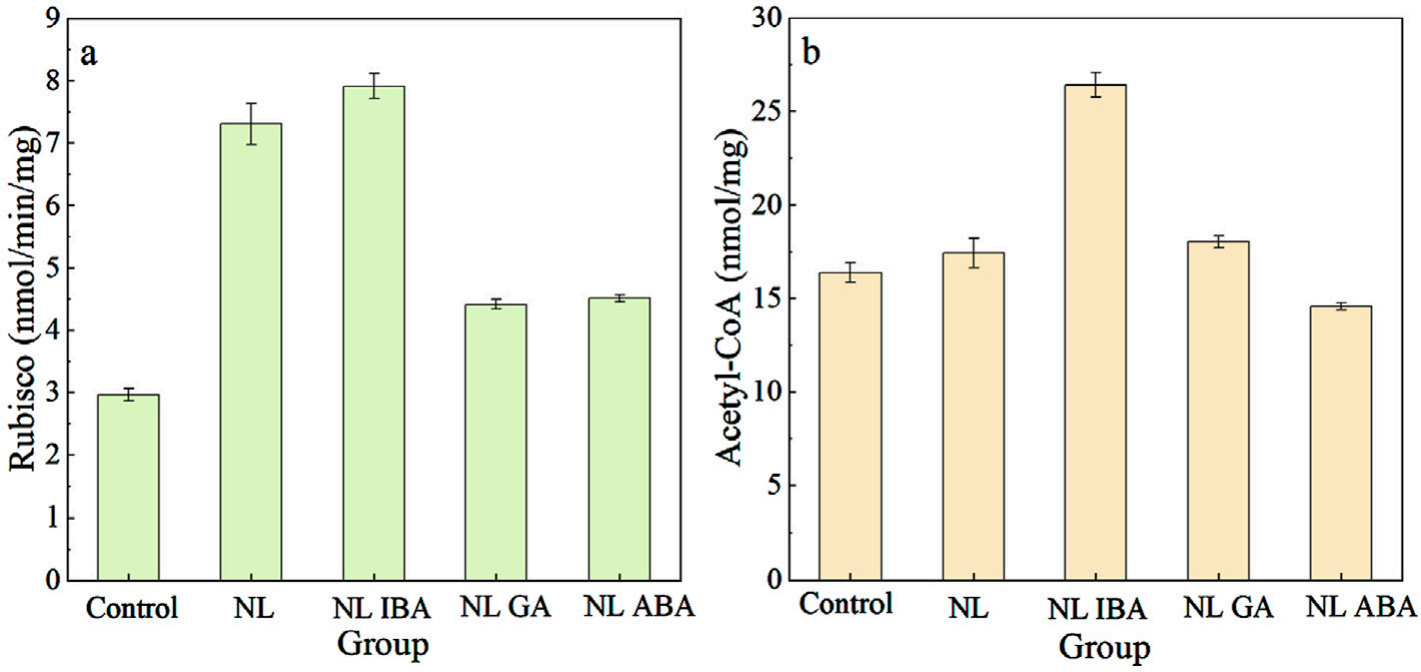
Changes of Rubisco activity **(a)** and Acetyl-CoA **(b)** content under different conditions.

Ribulose-1,5-bisphosphate carboxylase/oxygenase (Rubisco) was a key enzyme in photosynthesis, and played a role in carbon fixation and photorespiration (Wan et al., 2011; Zhang et al., 2016). As shown in Figure 3a, the Rubisco activity of microalgae was increased under nitrogen stress, compared with control. As we known that nitrogen limitation would inhibit the nitrogen assimilation process. Previous study showed that photorespiration was important for nitrogen assimilation (Rachmilevitch and Bloom, 2004). Huang et al. (2015) also proved that under nitrogen stress, the photorespiration pathway was activated to promote amino acid metabolism. Therefore, the enhanced Rubisco activity may indicate that the photorespiration pathway has been activated under nitrogen limited condition. The explanation of this result might be that photorespiration of microalgae cells was enhanced to adapt the adverse environmental conditions (Leon-Vaz et al., 2021). Under this condition, photorespiration could prevent the thylakoid membrane from producing excess electrons in the reduced state by providing sufficient electron acceptors for PSI, thereby improving the oxidative damage resistance of microalgae. Under NL treatment, adding phytohormones reduced the activity of Rubisco. Among them, the supplement of IBA had no significant effect on Rubisco activity (P > 0.05). While the Rubisco activities of GA and ABA group were 4.41 ± 0.08 and 4.51 ± 0.06 nmol/min/mg, which decreased by 39.59% and 38.22% (p < 0.05). This may be because that the supplement of phytohormones alleviated the oxidative damage of microalgae cells and weakened photorespiration, directing more energy flow to microalgae growth. This result was also consistent to the higher biomass shown in Figure 1. On the other hand, different phytohormones have different regulatory functions, which required further in-depth research in the future.

As one of the important intermediate metabolites, Acetyl-CoA could participate in tricarboxylic acid (TCA) and glyoxylate cycle, a process related to microalgae growth and cell composition (Singh et al., 2025a; Sun et al., 2020). As shown in Figure 3b, the Acetyl-CoA content in NL group reached 17.41 ± 0.79 nmol/mg, which was higher than that in the normal nitrogen concentration group (16.37 ± 0.53 nmol/mg). As we known, under nitrogen limited condition, the level of oxidative stress within the cells would rise, which in turn promoted the accumulation of lipids to protect the cells (Nagappan et al., 2020). While the synthesis of lipids required the participation of Acetyl-CoA, and Acetyl-CoA could be generated through glycolysis metabolism. Therefore, in this study, the synthesis of Acetyl-CoA increased under nitrogen limited conditions. Besides, microalgae activated glycolysis pathway not only promoting the Acetyl-CoA synthesis, but also providing energy basis for resistance to stress condition (Li et al., 2023; Verma et al., 2021). Phytohormones could also affect the Acetyl-CoA activity of *D. salina* under nitrogen stress. Under nitrogen limited treatment, the supplement of ABA decreased Acetyl-CoA content, while IBA and GA increased the Acetyl-CoA content. This might be related to the fact that different phytohormones exert different functions. For instance, ABA could promote catabolic processes, which may inhibit the synthesis of Acetyl-CoA (Liu L. et al., 2024). While IBA and GA could enhance glycolysis pathway and promote the conversion of glucose into pyruvate (Quan et al., 2017; Yu et al., 2016), and then pyruvate undergoes metabolism to generate Acetyl-CoA. To sum up, these results suggested that the exogenous phytohormones affected the carbon metabolism of *D. salina* cells under nitrogen stress condition, and different phytohormones might play a different role.

### 3.4 Response of antioxidant and nonenzymatic antioxidant on phytohormones and nitrogen stress

**FIGURE 4:**
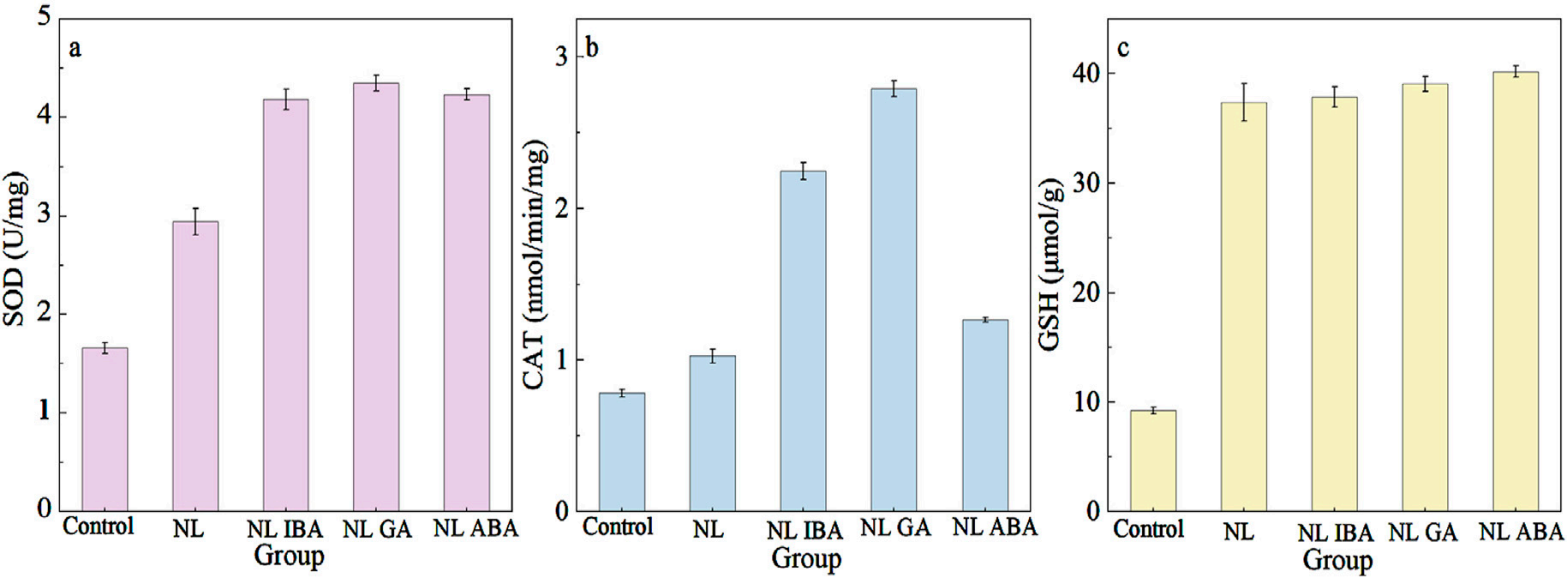
Effect of different phytohormones on **(a)** SOD activity, **(b)** CAT activity, **(c)** GSH activity of *Dunaliella salina* after 7-day cultivation.

The intracellular reactive oxygen species (ROS) in microalgae would be increased exposed to abiotic stress, which could cause oxidative damage to microalgae cells (Zhao et al., 2019a; Zhou and Gao, 2023). In response to this environmental stress, the antioxidant system in microalgae would be activated to alleviate the intracellular oxidative damage and haste the establishment of cellular homeostasis (Zhou et al., 2024). Antioxidant enzymes such as superoxide dismutase (SOD) and catalase (CAT), and nonenzymatic antioxidant such as glutathione (GSH) belong to the antioxidant system and played a role in protecting cells (Zhu et al., 2019). Previous study has indicated that nitrogen stress could cause cell oxidate stress condition and activate the antioxidant system (Yu et al., 2018). The addition of phytohormones could help to resist oxidative stress (Song et al., 2022). SOD converts superoxide radical (O^2−^) of ROS to hydrogen peroxide (H_2_O_2_) and O_2_, thereby preventing the production of superoxide anion radicals (Xiong et al., 2017). As shown in Figure 4a, nitrogen stress significantly increased SOD activity compared with the normal nitrogen group. The SOD activity of the microalgae cells in the IBA, GA and ABA treatment groups was 1.42 times, 1.48 times and 1.44 times that of the NL group, respectively. The results were similar to the previously reported observations on the upregulation of SOD activity with exogenous melatonin under nitrogen starvation culture (Zhao et al., 2018). Compared with the normal system, the CAT activity of the group exposed to nitrogen stress was increased. The phytohormones treatment further promoted the enzyme activity, which showed a similar trend with SOD activity with phytohormones. In addition, the CAT activity (Figure 4b) of microalgae under nitrogen limited combined with GA treatment was the highest among all the groups, and it was 2.71 times higher than in the NL group. Moreover, an obvious increase of GSH content was observed under nitrogen stress (Figure 4c). The GSH content of microalgae exposed to nitrogen stress condition was 4.06-fold higher than of the control. And the addition of exogenous phytohormones did not change the GSH content significantly. These results indicated that phytohormones might increase the antioxidant enzyme activity to eliminate the excess ROS and assist microalgae response to nitrogen stress. It was also consistent with the previously observed effects of exogenous phytohormones on *Chlorella* exposed to seawater stress (Zhou et al., 2024). These results showed that phytohormones could alleviate microalgae cells oxidant stress by further activating the antioxidant systems of microalgae under adverse environmental conditions. In addition, exogenous phytohormones played a role in promoting the growth of microalgae cells, accelerating the establishment of cell homeostasis and improving the cells tolerance to abiotic stress in CAMC system.

### 3.5 Microalgal primary metabolite accumulation after exposure to phytohormones and nitrogen stress

**FIGURE 5:**
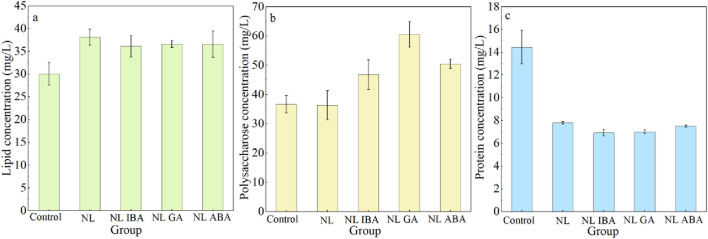
The primary metabolites accumulation of *Dunaliella salina* after 7-day cultivation under different conditions: **(a)** lipid; **(b)** polysaccharose; **(c)** protein.

Microalgae could fix CO_2_ through photosynthesis and convert it into metabolites such as lipid, protein and carbohydrates (Ruiz et al., 2016). The change of culture conditions could affect the carbon flow in microalgae cells, and then affect the accumulation of metabolites. The changes of lipid, polysaccharose and protein accumulation of microalgae in different treatments were depicted in Figure 5. An obvious enhancement of lipid yield was observed under nitrogen stress. The maximum lipid productivity of microalga reached 38.10 ± 1.77 mg/L, which was obtained in *D. salina* cells exposed to nitrogen stress, and it was 26.92% higher than that of the control group, respectively. The results were aligned with the previous studies which reported that the lipid content of microalgae was increased by nitrogen starvation treatment (Dahmen-Ben Moussa et al., 2019; Renuka et al., 2017; Wei et al., 2022). The explanation of the results was that the synthesis of neutral lipid was promoted to protect microalgae cells from oxidative damage. Previous also reported that the expression of key enzymes related to the lipid synthesis in microalgae was upregulation under nitrogen stress (Singh et al., 2017). Moreover, the addition of phytohormones did not significantly affect the lipid accumulation of microalgae on the basis of nitrogen stress treatment. Compared with the lipid content of microalgae obtained in nitrogen stress treatment without phytohormones, a slight decrease of lipid yield was observed in combined nitrogen stress treatment and phytohormones, which was still higher than that of normal system. This decrease may be because that phytohormones alleviated the oxidative stress of microalgae cells under adverse environmental conditions, which in turn affected the lipid content. The results in this study were different from the previous study which reported that the supplement of strigolactone further improved the lipid content of *Monoraphidium.sp* under nitrogen stress (Song et al., 2020), which may be explained by the different adaptability of different algae sepsis to nitrogen stress and different responses to phytohormones.

The carbon flow reallocation of microalga cells due to nitrogen stress and phytohormones induced the change of polysaccharose content. As shown in Figure 5b, phytohormones promoted the polysaccharose accumulation under nitrogen stress, which was similar to the changes of lipid content. The polysaccharose concentrations in IBA, GA and ABA group reached 46.74 ± 5.00 mg/L, 60.51 ± 4.30 mg/L and 50.44 ± 1.56 mg/L, respectively, which were 28.84%, 66.78% and 39.02% higher than that in nitrogen limited group. It could be observed that the effect of GA on polysaccharose accumulation was the most significant, the concentration of GA group was 1.65- fold than that of the control (*Dunaliella* medium). The change trend of protein was opposite to that of polysaccharide (Figure 5c), and the protein content of microalgae in nitrogen limited medium was lower than that of control group (p < 0.05). This may be because that protein synthesis was inhibited due to the lack of nitrogen content under nitrogen stress, thus carbon flow was directed to lipid and carbohydrates. Previous also reported that the protein content was reduced under nitrogen starvation (Yilancioglu et al., 2014). Moreover, the addition of phytohormones further decreased the protein content of *D. salina* exposed to nitrogen limited condition. This also corresponded to the fact that phytohormones promoted the synthesis of polysaccharide. In this study, nitrogen stress affected the carbon metabolism of microalgae cells, with more carbon flow directed to lipid and polysaccharose synthesis, while protein synthesis was inhabited. Similarly, the synthesis of carbohydrate and lipid was enhanced to help microalgae cells to resist the abiotic stress (Zhang et al., 2023). In addition, the supplement of GA further affected the direction of carbon flow under nitrogen stress, and the direction of carbon flow to polysaccharose was further strengthened, inducing the higher polysaccharose accumulation in CAMC system.

### 3.6 Phytohormones combined with nitrogen stress promoted β-carotene synthesis

**FIGURE 6:**
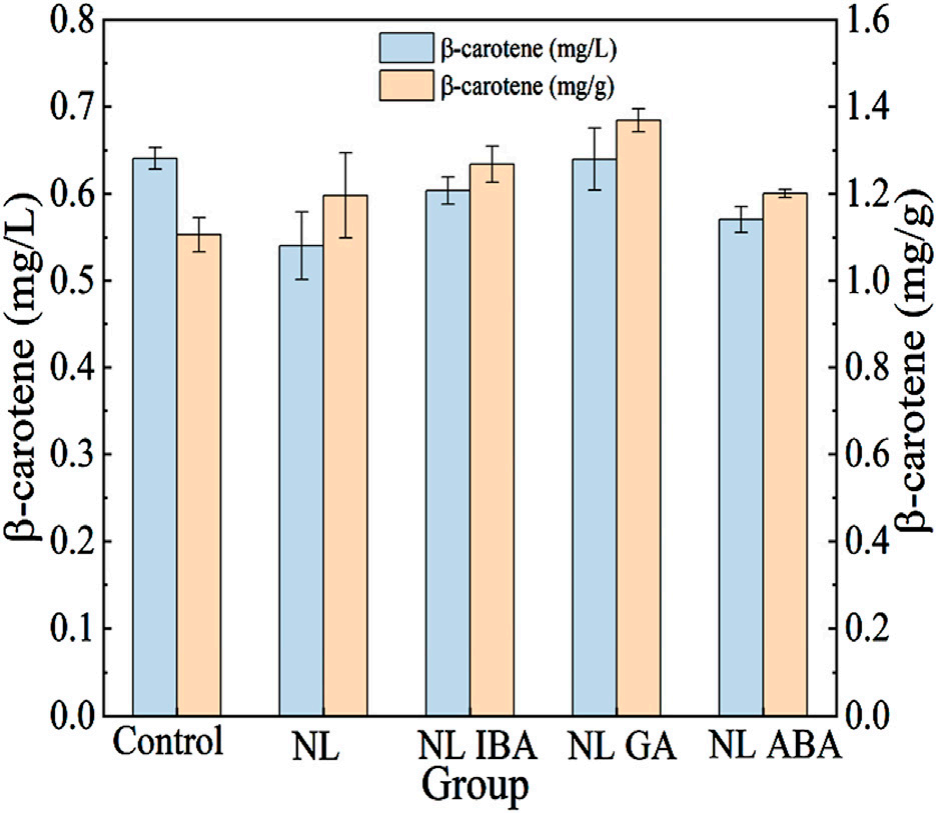
Changes of β-carotene after cultivation under different conditions.

As shown in Figure 6, the β-carotene yield (mg/L) and content (mg/g cell dry weight) of *D. salina* cells was measured at the end of the culture. It could be observed that nitrogen stress had a positive impact on the accumulation of β-carotene in microalgae cells. Although the β-carotene yield in nitrogen limited microalgae cells was lower than that in the normal microalgae cells, which might due to the inhibitory effect of nitrogen stress on microalgae growth, the β-carotene content was obviously increased. The results were agreement with the previously reported observation on the β-carotene content in *D. salina* cells was increased under nitrogen starvation condition (Xi et al., 2020). In addition, the microalgae cells would produce excess ROS in response to nitrogen stress, which might induce the accumulation of β-carotene. ROS could regulate the key enzymes involved in β-carotene synthesis, and a positive correlation of intracellular ROS and β-carotene content under stress condition was observed (Xi et al., 2020). Previous also reported the content of β-carotene in *D. salina* was increased in response to high light intensity combined molybdenum disulfide nanoparticles stress (Luo et al., 2021).

Phytohormones combined with abiotic stress was an effective way to produce high-value products, as shown in Table 1. In this study, it was worthy that phytohormones further promoted the accumulation of β-carotene under nitrogen stress treatment. For nitrogen limited condition, the addition of IBA, GA and ABA improved the content and yield of β-carotene. Among them, the effect of GA was the most significant, the concentration of β-carotene supplemented with GA was 18.52% higher than that without GA in NL group. Moreover, the β-carotene content was promoted by 14.46% under GA treatment in NL group. The results suggested that the supplement of phytohormones combined nitrogen limited treatment could be a promising strategy to product β-carotene in *D. salina* in CAMC system.

**TABLE 1:** Comparative study on the application of phytohormones in microalgae.

Order	Algae species	Phytohormones	Products	Change	Reference
1	*Chlorella vulgaris*	Indole-3-acetic acid	lipid	Increased by 16%	Chang et al. (2022)
2	*Spirulina platensis*	Melatonin	phycocyanin	Increased by 13.74%	Li et al. (2024)
3	*Acutodesmus obliquus*	Zeatin	lipid	Increased by 64.95%	Renuka et al. (2017)
4	*Dunaliella salina*	Indole-3-acetic acid	Lipid	1.42 times	Singh et al. (2024b)
5	*Dunaliella bardawil*	Melatonin	Lutein	1.24 times	Xie et al. (2022)
6	*Dunaliella salina*	Gibberellin	β-carotene	Increased by 18.52%	This study

## 4 Conclusion

This work evaluated the response of *D. salina* to nitrogen stress in CAMC system, and explored the effect of different phytohormones on microalgae growth and metabolism under nitrogen stress. The results showed the addition of phytohormones could alleviate oxidative stress to counteract the negative impact of nitrogen stress on the growth of microalgae. Moreover, adding GA promoted pigment synthesis and enhanced photosynthetic activity. The changes of Rubisco activity and Acetyl-CoA content illustrated that GA further affected the carbon metabolism of microalgae cells. The nitrogen limited treatment combined with GA effectively improved the β-carotene content of *D. salina* in CAMC system. This study provided guidance for the utilization of phytohormones to promote economically valuable metabolites, and was also conducive to the application of CAMC. In the future, transcriptomics could be combined with Q-PCR technology to further reveal the pathway that phytohormones regulated carbon metabolism and β-carotene synthesis under nitrogen stress. Besides, life cycle assessment and techno-economic analysis could be used to explore the economic and environmental feasibility of applying phytohormones.

## Data Availability

The original contributions presented in the study are included in the article/supplementary material, further inquiries can be directed to the corresponding authors.
